# RSV infection disrupts gut microbiota and metabolic homeostasis in mice, regulating pulmonary inflammation via the SPHK/S1P pathway

**DOI:** 10.1128/spectrum.03035-24

**Published:** 2025-08-14

**Authors:** Ruo-gu Yu, Xue-mei Li, Li Zhang, Jing Jiang, Bin Zhang, Xiao-bin Wu

**Affiliations:** 1 Chongqing Health Center for Women and Children 117720 , Chongqing, China; 2 Women and Children's Hospital of Chongqing Medical University 117720, Chongqing, China; 3 Department of Dermatology, Chongqing Traditional Chinese Medicine Hospital/The First Affiliated Hospital of Chongqing College of Traditional Chinese Medicine 711051 , Chongqing, China; University of Arkansas Fayetteville, Fayetteville, Arkansas, USA

**Keywords:** RSV, bronchiolitis, gut microbiome, sphingolipids

## Abstract

Respiratory syncytial virus (RSV) is a primary pathogen for lower respiratory tract infections in children, posing a significant health threat. However, the systemic effects of RSV, particularly on gut microbiota and immune regulation along the gut-lung axis, are not well understood. We utilized a mouse model of RSV infection and assessed dynamic changes in the gut microbiome via high-throughput sequencing. We also investigated the interplay between pulmonary inflammation and gut microbiota, as well as their metabolites, through metabolomics analysis. RSV infection did not substantially alter the gut microbiota’s alpha diversity but modified the relative abundance of specific phyla and genera. Notably, there was an increase in taxa such as Bacteroidetes and Veillonella, which may be linked to inflammation. Concurrently, sphingolipid components, including sphingomyelin, sphingosine, and ceramide, were significantly reduced in RSV-infected mice, correlating with increased pulmonary sphingosine-1-phosphate (S1P) protein expression. Receiver operating characteristic analysis indicated the potential of sphingolipids as biomarkers for distinguishing healthy from RSV-infected states. Inhibition of the S1P metabolic pathway, using sphingosine kinase (SPHK) and S1P inhibitors, reduced S1P expression and pulmonary inflammation, as well as pro-inflammatory cytokines like interleukin-1 beta, interleukin-6, and tumor necrosis factor-alpha. Dietary changes, notably a low-fat diet, ameliorated lung inflammation and neutrophil accumulation in bronchoalveolar lavage fluid, highlighting the role of dietary intervention in managing RSV infection. Immunophenotyping revealed the effects of SPHK and S1P inhibitors on lymphocyte subpopulations, foreshadowing their roles in modulating immune responses. These findings offer novel insights into how RSV infection modulates pulmonary inflammation by altering gut microbiota and metabolic pathways, providing a basis for new therapeutic strategies.

**IMPORTANCE:**

Our research provides new insights into how respiratory syncytial virus (RSV) infection affects the host's gut microbiota, lipid metabolism, and the immune-inflammatory network. The findings demonstrate that dietary modulation and pharmacological intervention of the sphingosine-1-phosphate pathway can mitigate inflammation caused by RSV infection, presenting potential avenues for the development of novel therapeutic strategies to treat RSV infections.

## INTRODUCTION

Respiratory syncytial virus (RSV) is a prevalent respiratory pathogen, particularly causing upper respiratory tract infections and pneumonia among infants and children (1). While the precise mechanisms of lung disease caused by RSV remain unclear (2), inevitable metabolic byproducts of the gut microbiota may play a role in the progression of respiratory illnesses (3). Recent studies suggest that sphingolipids may be implicated in the pathogenesis of bronchiolitis (4), and our previous research has indicated an increase in intestinal sphingolipid metabolism in children with bronchiolitis (5). As a crucial component of cell membranes, sphingolipids not only sustain cell structure and function but also are involved in intercellular signaling (6); however, the mechanisms by which they modulate bronchiolitis are not well understood. The gut-lung axis, a bidirectional communication network between the gastrointestinal and respiratory systems, has emerged as a critical regulator of immune homeostasis and host defense against infections (7, 8). Recent studies indicate that gut microbiota-derived metabolites, such as short-chain fatty acids (SCFAs) and sphingolipids, can modulate pulmonary immune responses through systemic circulation or vagus nerve signaling (9). For instance, SCFAs enhance anti-viral immunity by promoting dendritic cell maturation and T-cell differentiation (10), while dysbiosis of gut microbiota is linked to exacerbated inflammation in respiratory viral infections (9). In the context of RSV, however, the role of gut-lung crosstalk remains underexplored. This study aims to establish an RSV-infected mouse model to assess fecal gut microbiota and lipidomic profiles, analyzing the association between RSV-induced pulmonary infections and the gut microbiota as well as their metabolic products—lipids.

## MATERIALS AND METHODS

### Establishment of the RSV model

(i) Mice preparation: BALB/c mice, SPF grade, of both sexes, aged 6–8 weeks, provided by the Animal Experiment Center of Chongqing Medical University, were housed in specific pathogen free (SPF) conditions with autoclave-sterilized feed and water. BALB/c mice were selected due to their susceptibility to RSV-induced bronchiolitis, mirroring key pathological features observed in human infants (11). This model exhibits analogous immune responses, including Th2-skewed inflammation and mucus hypersecretion, making it translational for studying RSV-host interactions (12). To minimize confounding effects from co-housing, mice were individually housed post-RSV inoculation. Twelve mice were randomly divided into experimental and control groups, with six in each, and were housed individually to prevent intestinal microorganism interaction. (ii) Hep-2 cell culture and RSV propagation: Hep-2 cells and RSV A2 strain were acquired from ATCC. Post 90% confluence in a culture flask, RSV was inoculated, the old solution was discarded, replaced with Dulbecco’s Modified Eagle Medium containing 10% fetal bovine serum, and incubation commenced at 37°C with 5% CO_2_. Syncytium injuries were monitored until cell fusion reached 90%–100%. Subsequently, viral fluid was collected and centrifuged at 4°C, 12,000 rpm for 10 minutes, with the supernatant quickly transferred to −80°C for storage. The titer of used RSV A2 was 1.5 × 10^8^ PFU/mL, confirmed by plaque assay. (iii) Animal model development for RSV infection (mouse bronchiolitis): female BALB/c mice aged 6–8 weeks were divided between RSV-infected and control groups. The mice were euthanized on day 5, and lung specimens were collected. The method of euthanasia used in our study for mice was cervical dislocation performed by personnel specifically trained for this procedure.

### Model group treated with an S1P/SPHK inhibitor

Twenty female BALB/c mice were randomly assigned via computer-generated randomization into four experimental groups: Naïve control, RSV infection model, RSV model + SPHK inhibitor, and RSV model + sphingosine-1-phosphate (S1P) inhibitor. Following RSV model establishment, mice in the inhibitor treatment groups received daily intraperitoneal injections of the respective SPHK (SKI II, 10 mg/kg, once daily) or S1P (PF429242, 10 mg/kg, once daily) inhibitor for three consecutive days, whereas control animals were administered equivalent volumes of sterile saline. All mice were housed under identical specific pathogen-free conditions with *ad libitum* access to standard chow and water.

### Fecal collection and processing

Fecal samples were collected on the day of euthanasia and preserved at −80°C. Samples were sent to Personalbio company in Shanghai for 16S rRNA sequencing and lipidomic analysis.

### Bronchoalveolar lavage fluid cell count

After cervical dislocation, mice were secured dorsally to expose the trachea and bilateral lungs. The left lung was tied and intubated for lavage with 0.5 mL precooled phosphate-buffered saline (PBS) twice. The recovered lavage was centrifuged at 4°C, 2,500 rpm/min for 5 minutes, and the cell pellet was stained with Wright’s stain for differential cell counting.

### Lung histopathology section processing

(i) Specimen fixation, dehydration, and embedding: fresh left lung tissue was fixed with 10% neutral formalin for over 24 hours, rinsed overnight, dehydrated for 8–10 hours, then embedded in paraffin to create tissue blocks. (ii) Sectioning and staining: the blocks were cut, placed on slides, and heated to melt paraffin, followed by hematoxylin and eosin (HE) staining, with specific steps being outlined. (iii) Histomorphometric analysis: inflammation assessment was based on the scoring method by Myou et al. (13) to evaluate lung histopathological changes.

### High-fat and low-fat diet feeding

Mice were fed with a 60% high-fat or 3% low-fat diet provided by Jiangsu Xietong Biotechnology Co., Ltd., product code XT19008.

### Immunofluorescence staining of mouse lung tissues

(i) Deparaffinization: newly baked paraffin sections were deparaffinized and rehydrated. (ii) Antigen retrieval: pressure-cooked in citrate buffer. (iii) Washing and blocking: performed with PBS and normal donkey serum. (iv) Primary and secondary antibody incubation: application of primary antibodies (CD19, Abcam: ab245235, 1:200; CD49b, Abcam: ab185548, 1:200; CD3, Abcam: ab135372, 1:200) followed by fluorescent-conjugated secondary antibodies (Abcam, ab150081, 1:400). (v) Nuclear staining with 4′,6-diamidino-2-phenylindole (DAPI) and mounting: slides were stained with DAPI and covered with an anti-fade mounting medium. (vi) Cell counting: positive cells were counted under a microscope. (vii) Image source clarification: the “Mock + Normal” IF images in Fig. 10A and 11A were randomly selected from consecutive sections of the same mouse cohort (6 mice/group, 1–2 sections/mouse). Technical similarities may occur due to proximal tissue sampling.

### Protein extraction from mouse lung tissue

(i) Samples were collected post-euthanasia. (ii) Samples were homogenized. (iii) Homogenates were lysed in radioimmunoprecipitation assay buffer containing protease and phosphatase inhibitors. (iv) Lysis was performed on ice. (v) Samples were centrifuged to remove debris, and the supernatant was used for protein determination.

### Western blot procedure

(i) Protein extraction and quantification were performed using lysis buffer with protease and phosphatase inhibitors. (ii) Electrophoresis, transfer to PVDF membranes, and blocking with skim milk. (iii) Incubation with primary antibodies (Anti-S1P, Abcam: ab140592, 1 µg/mL; Anti-SPHK1, Cell Signaling Technology: #12071, 1:500; Anti-IL-6, Invitrogen: 4H16L21, 0.5 µg/mL; Anti-IL-1β, Abcam: ab283818, 1:1,000; Anti-TNF-α, Abcam: ab183218, 1:1,000) and appropriate secondary antibodies (Abcam, ab205718, 1:2,000). (iv) Imaging was conducted using an imaging scanner, with quantitative analysis by Image J software.

### Statistical analysis

Continuous data are presented as mean ± standard error of the mean (SEM). The normality of distribution was assessed using the Shapiro-Wilk test, and homogeneity of variance was confirmed via Levene’s test. For normally distributed data with equal variance, comparisons between groups were performed using one-way analysis of variance (ANOVA) followed by Tukey’s *post hoc* test for multiple comparisons. Non-normally distributed data, including alpha diversity indices (Shannon, Simpson, and Chao1), were analyzed using the non-parametric Mann-Whitney *U* test for two-group comparisons or the Kruskal-Wallis test with Dunn’s correction for multi-group comparisons. Categorical variables were evaluated using the chi-square test. Beta diversity was assessed via principal coordinate analysis (PCoA) based on Bray-Curtis dissimilarity, and permutational multivariate analysis of variance with 999 permutations was applied to test group differences. To identify microbial taxa with discriminatory power between groups, random forest analysis (500 decision trees) was performed, selecting features with a mean decrease accuracy >2.0. The diagnostic potential of lipid metabolites (sphingomyelin [SM], sphingosine [SPH], and ceramide [Cer]) was evaluated using receiver operating characteristic (ROC) curve analysis, with an area under the curve (AUC) >0.7 considered indicative of diagnostic significance. Statistical significance was set at *P* < 0.05 for all analyses.

## RESULTS

### Analysis of alpha and beta diversity in two sample groups

**Fig 1:**
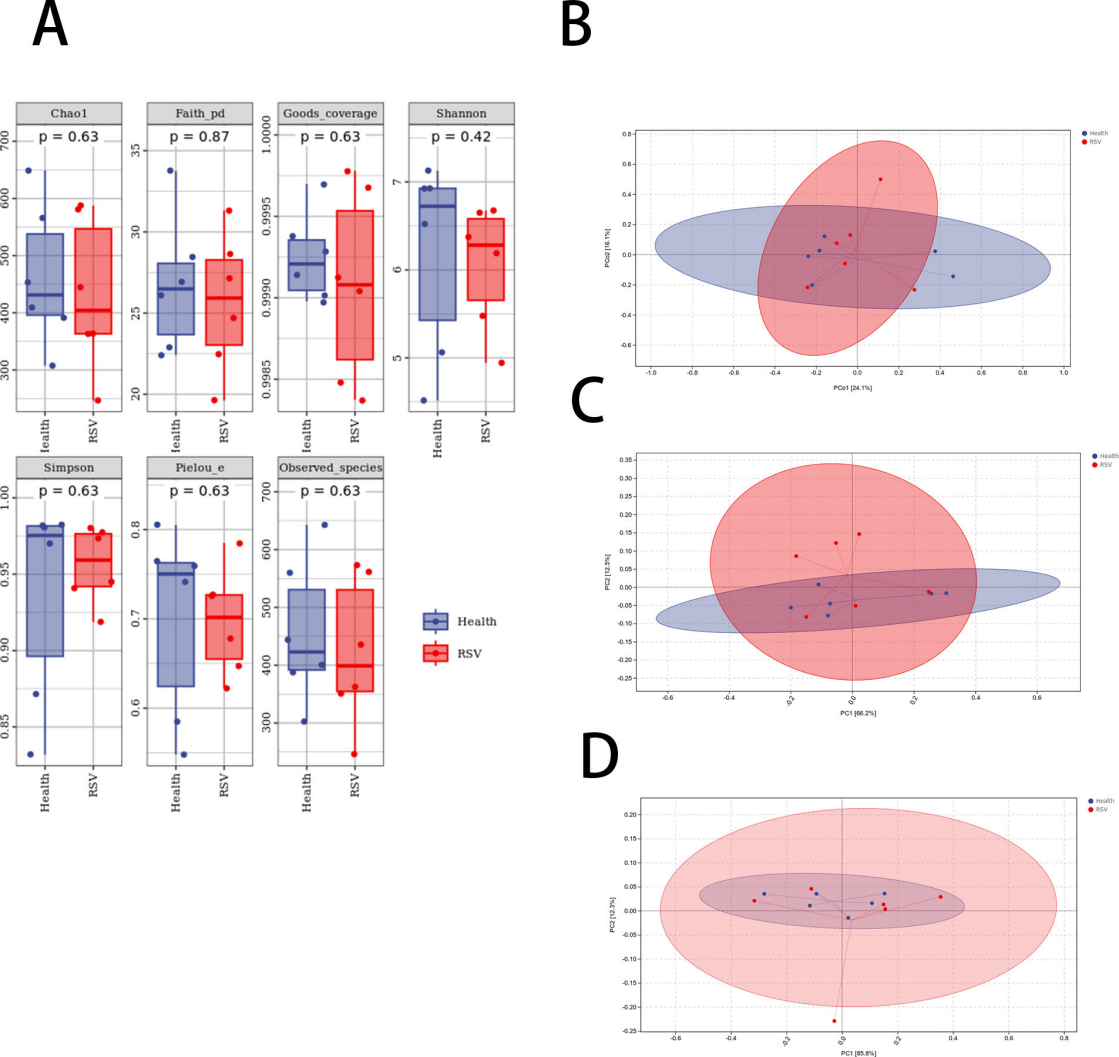
Analysis of gut microbiota diversity between groups. (A) Analysis of alpha diversity indices for gut microbial communities in both groups. (B) PCoA demonstrates overall microbial composition differences between the two sample groups. (C) Differences in microbial composition at the phylum level between the two sample groups. (D) Differences in microbial composition at the genus level between the two sample groups.

A differential analysis of alpha diversity in the gut microbiota of RSV infection model mice and healthy control mice was conducted using the Mann-Whitney *U* test. The results indicated no significant differences in alpha diversity between the two groups (Fig. 1A). PCoA was employed to assess the overall differences in the microbial composition between the two groups, while principal component analysis was used to analyze the differences at the phylum and genus levels between the two groups of samples. The results revealed that there were differences in the overall species composition of the gut microbiota between the RSV infection model mice and the healthy control mice (Fig. 1B), and both groups displayed differences in microbial composition at the phylum and genus levels (Fig. 1C and D).

### Dominant bacterial taxa at the phylum and genus levels in the two sample groups

**Fig 2:**
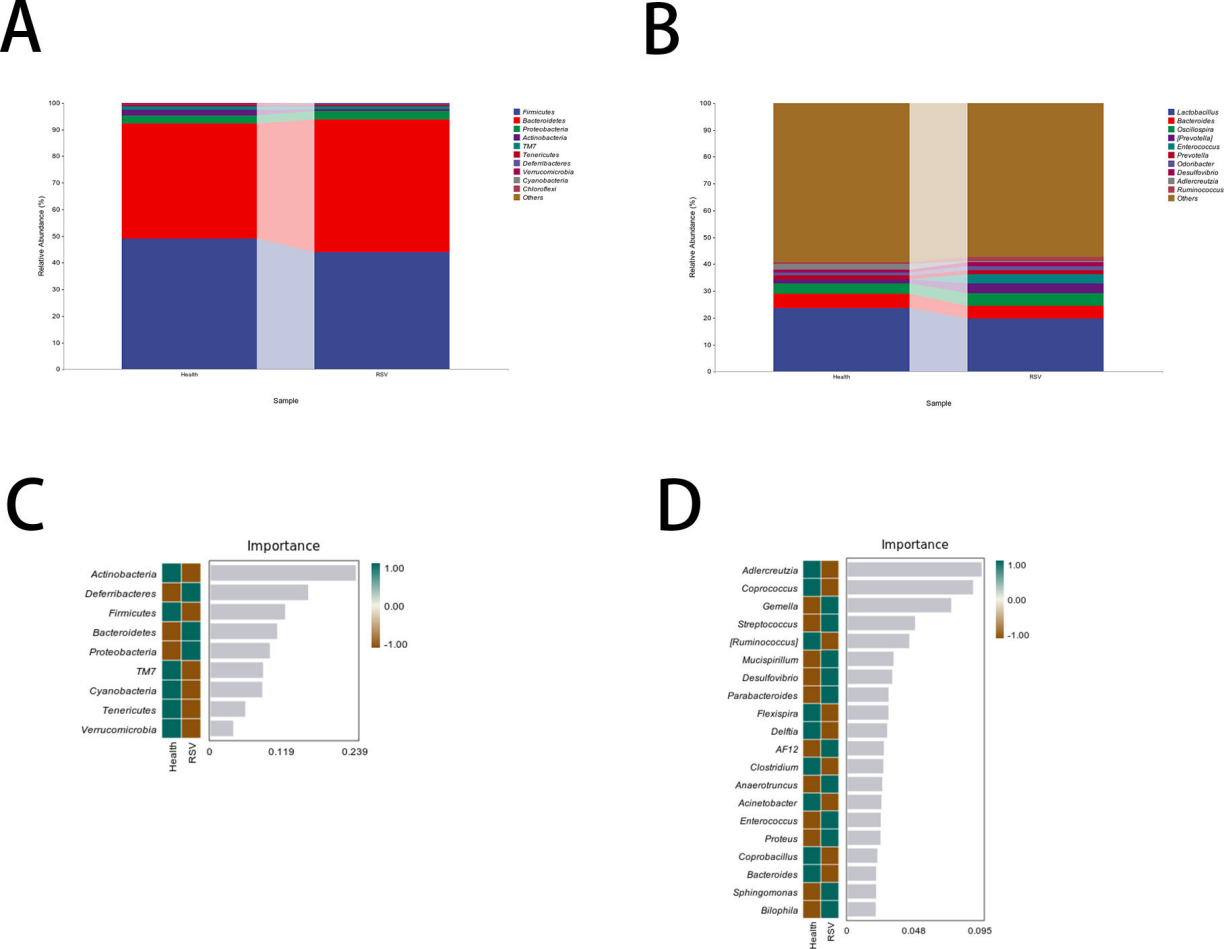
Changes and differences in microbial composition at different taxonomic levels and identification of key marker species. (A) Changes and differences in microbial composition at the phylum level. (B) Changes and differences in microbial composition at the genus level. (C) Random forest analysis identifying key marker species at the phylum level between the two groups. (D) Random forest analysis identifying key marker species at the genus level between the two groups.

Based on multivariate clustering analysis and random forest analysis at the level of bacterial operational taxonomic units, the following observations were made regarding the gut microbiota of RSV infection model mice compared to that of healthy mice. At the phylum level, there was an increase in Proteobacteria, Bacteroidetes, and Verrucomicrobia, whereas there was a decrease in Actinobacteria, Firmicutes, TM7 (a candidate phylum), Cyanobacteria, Tenericutes, and Planctomycetes. At the genus level, there was an increase in Geobacillus, Streptococcus, Helicobacter, Desulfovibrio, Parabacteroides, AF12 (an undefined genus), Anaerotruncus, Enterococcus, Bacteroides, Flavobacterium, and Alcaligenes, while there was a decrease in Adlercreutzia, Coprococcus, Cellulosilyticum, Campylobacter, Delftia, Clostridium, Acinetobacter, Faecalibacterium, and Prevotella (Fig. 2A through D). These shifts in specific bacterial phyla and genera could be relevant for understanding the impact of RSV infection on the gut microbiome and for identifying biomarkers or targets for intervention.

### Association network analysis

**Fig 3:**
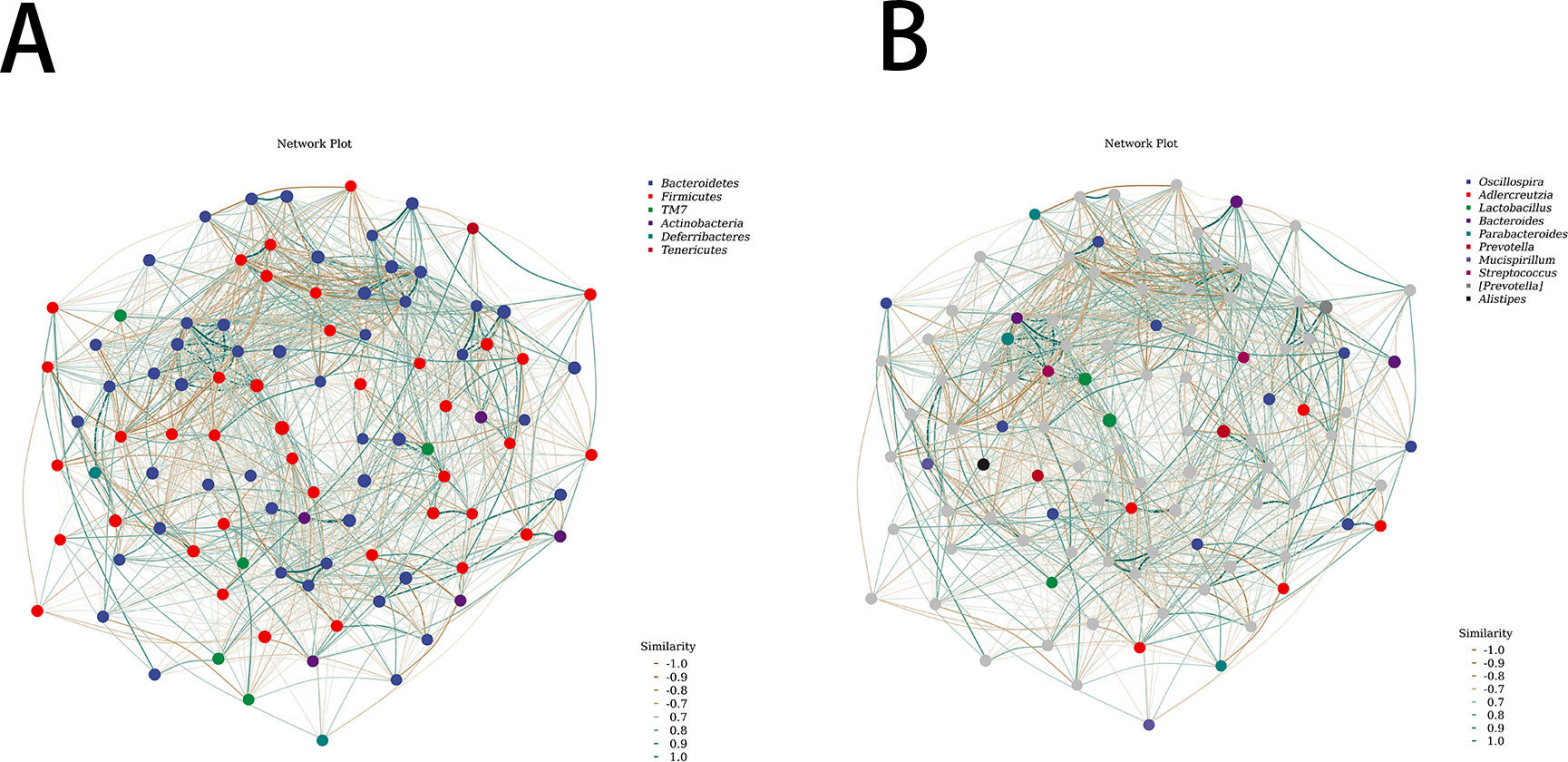
(A) Phylum-level association network analysis. (B) Genus-level association network analysis.

By conducting network analysis based on the mutual relationships among microbial members, we sought to identify key species that may be pivotal in influencing the composition changes within the entire community. The results suggest that at the phylum level, the key species that might serve as a leverage point for altering the community as a whole composition could be the Bacteroidetes phylum (Fig. 3A). At the genus level, the key species that might have a significant impact on community composition changes could be the genus Tremblaya (Fig. 3B).

### Lysosomal detection in murine feces

**Fig 4:**
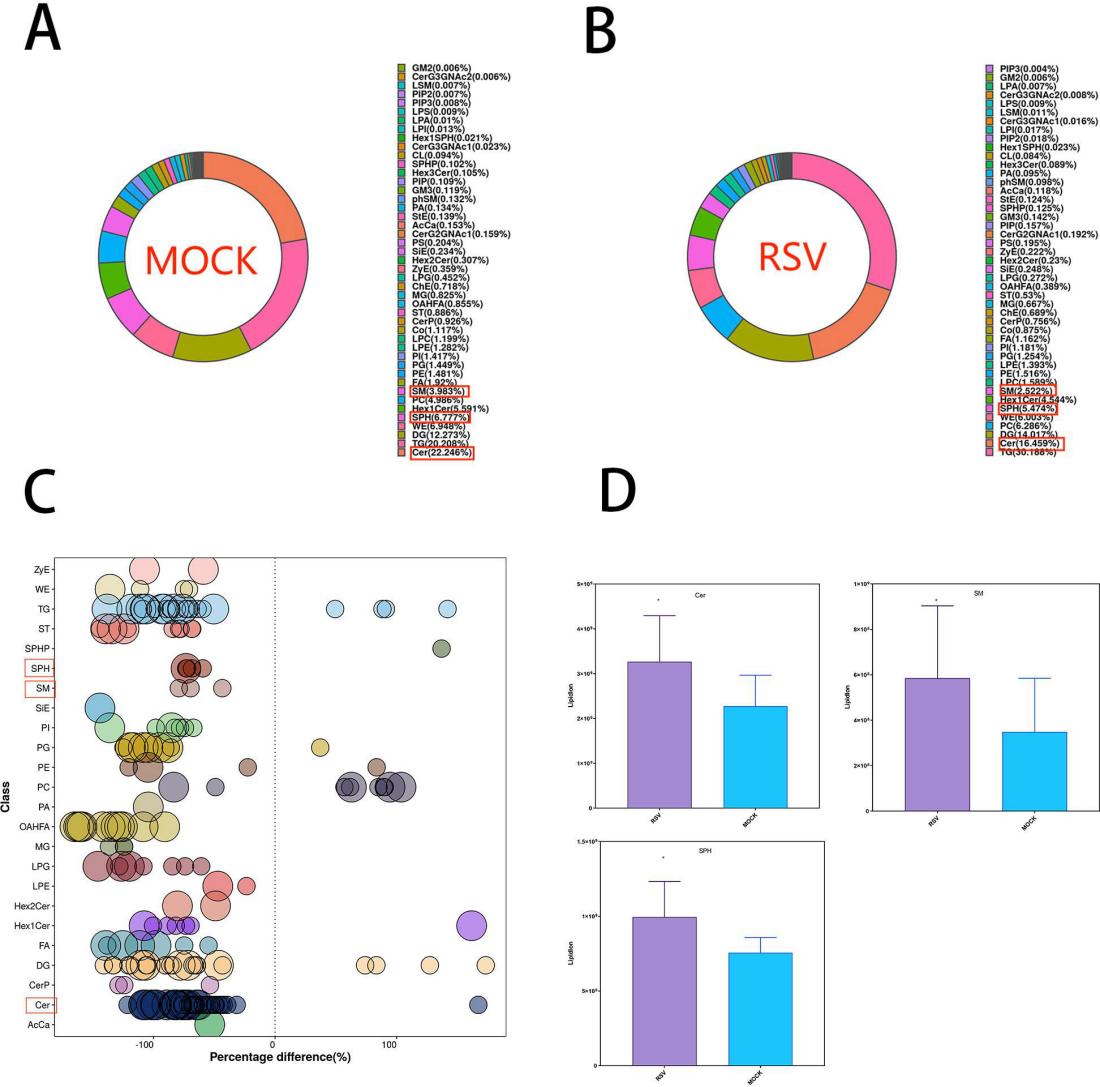
(A) Composition of fecal lipid subclasses in the RSV-infected mouse model. (B) Composition of fecal lipid subclasses in the healthy control group. (C) Lipid molecules with significant differences. (D) Comparative analysis of SM, SPH, and Cer levels between the two groups.

The lysosomal analysis on feces from mice with RSV infection model revealed significant reductions in the levels of major sphingolipid components associated with the generation of S1P. These components include SM, SPH, and Cer, as compared to the control group (Fig. 4A through D).

### ROC analysis

**Fig 5:**
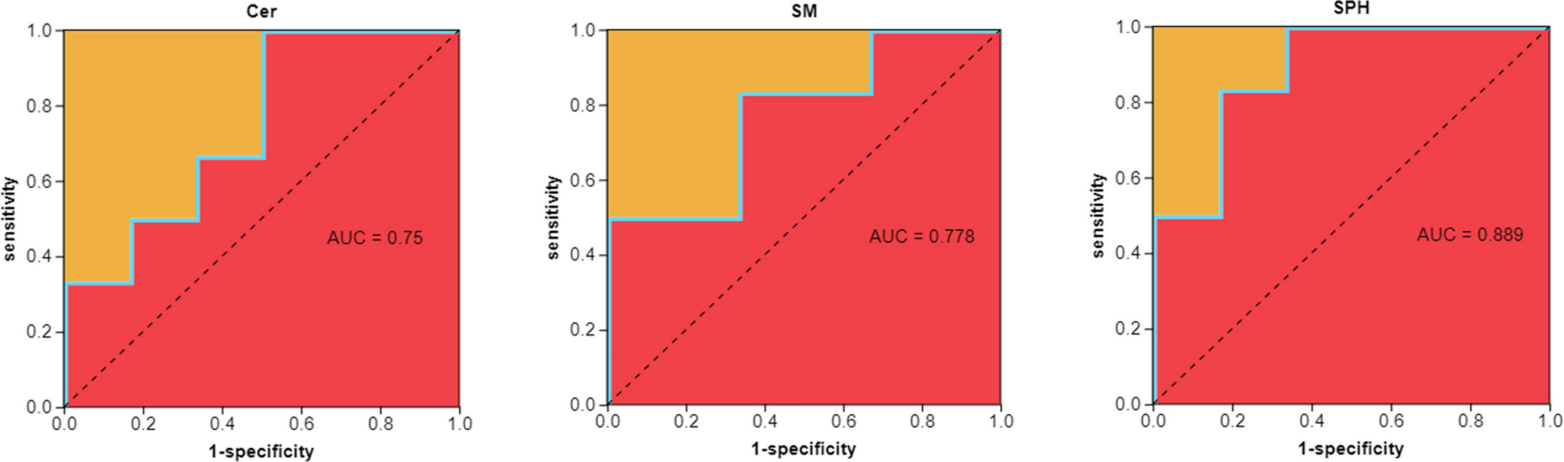
ROC analysis of potential biomarkers for distinguishing between healthy mice and mice infected with RSV.

The ROC analysis (AUC > 0.7) revealed that Cer, SM, and SPH could effectively differentiate between healthy mice and mice infected with RSV, as shown in Fig. 5.

### Western blot analysis

**Fig 6:**
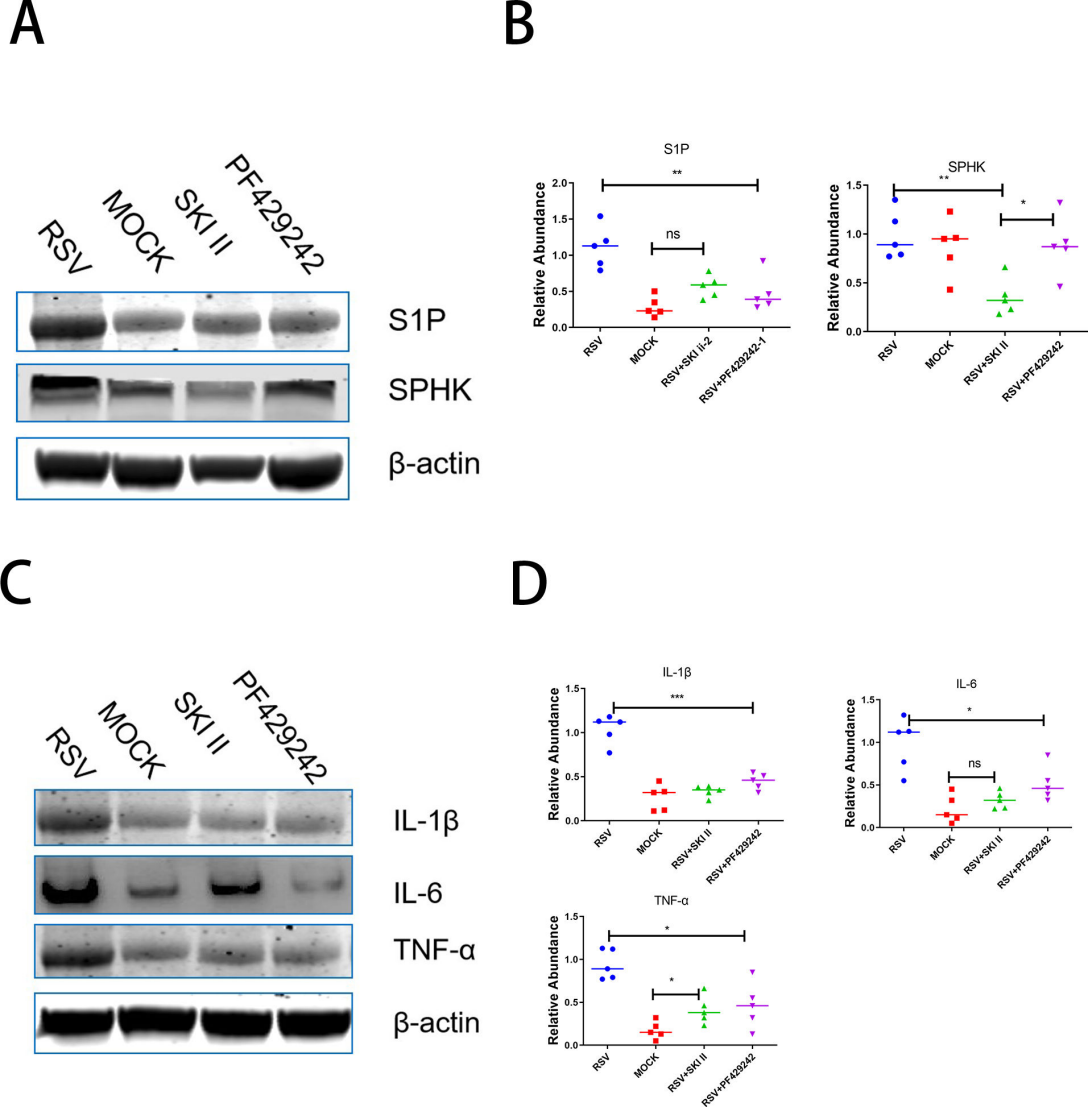
Analyses of protein expression and inflammatory factors. (A) Expression of S1P and SPHK proteins assessed via Western blot in RSV-infected mice, control group mice, RSV-infected mice treated with SPHK inhibitor, and RSV-infected mice treated with S1P inhibitor. (B) Quantitative comparison of S1P and SPHK protein levels across the four groups. (C) Western blot detection of inflammatory cytokines in the same four groups of mice. (D) Quantitative analysis of inflammatory cytokine levels across the four experimental groups. Significance levels: *P < 0.05, **P < 0.01, and ***P < 0.001. NS: not significant.

Western blot was employed to assess the expression of S1P, SPHK, and inflammatory cytokine proteins in the lung tissues of mice across different groups: the experimental group, the control group, the model group treated with an S1P inhibitor, and the model group treated with an SPHK inhibitor. The findings indicate that S1P protein levels were elevated in the lung tissues of RSV-infected mice compared to the control group. Furthermore, treatment with SPHK and S1P inhibitors in RSV-infected mice led to reduced expression of S1P protein in lung tissues, as seen in Fig. 6A and B. Similarly, levels of inflammatory cytokines interleukin-1 beta (IL-1β), interleukin-6 (IL-6), and tumor necrosis factor-alpha (TNF-α) were also downregulated, as demonstrated in Fig. 6C and D.

### Impact of diet on protein expression and inflammatory markers following RSV infection

**Fig 7:**
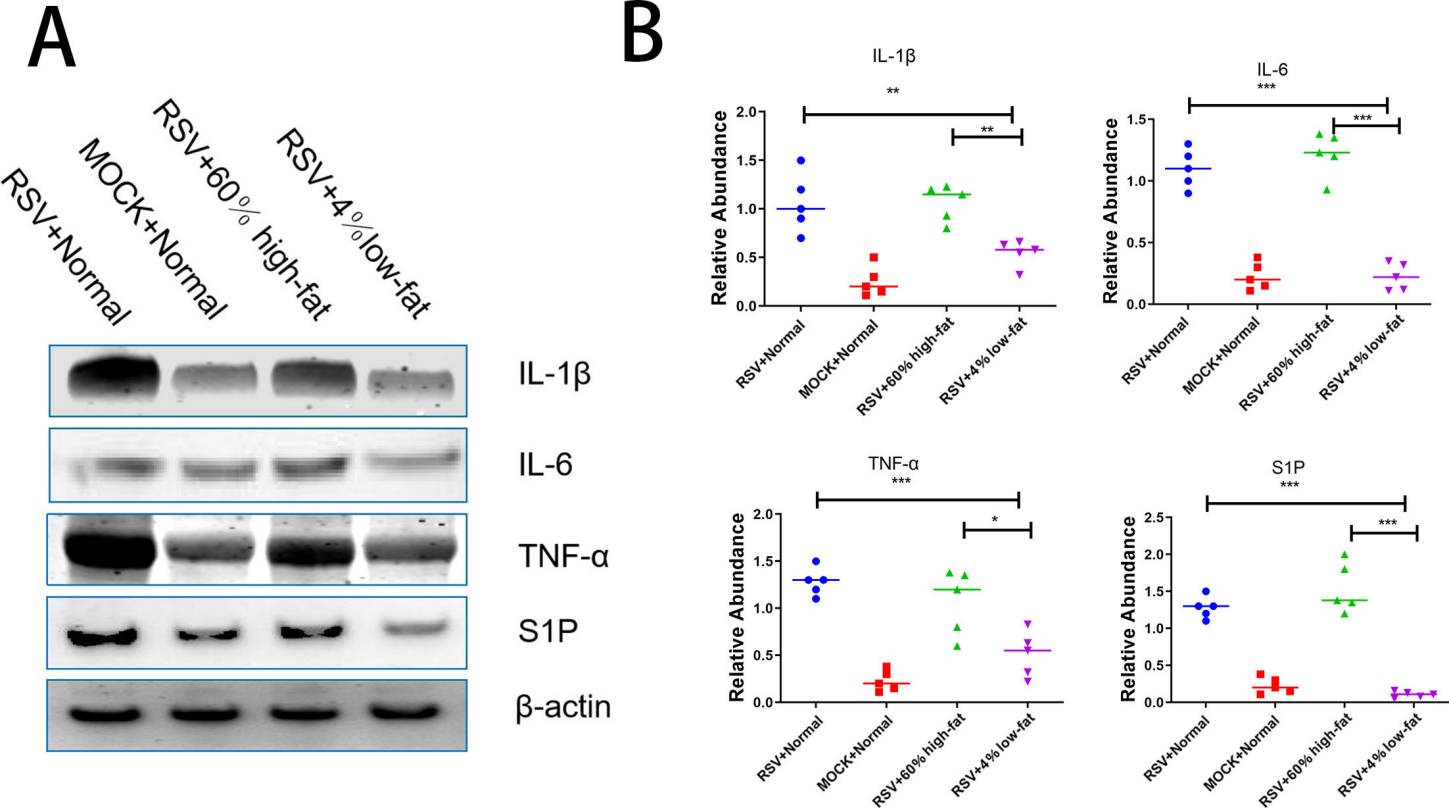
Analysis of S1P1 and inflammatory protein expression in response to dietary modulation post-RSV infection. (A) The Western blot analysis presents the differential expression of S1P1 and inflammatory cytokine proteins in various groups: RSV-infected mice, control group mice, RSV-infected mice fed a high-fat diet, and RSV-infected mice fed a low-fat diet. (B) Quantitative evaluation and comparison of S1P1 and inflammatory cytokine protein levels across the four groups. Significance levels: *P < 0.05, **P < 0.01, and ***P < 0.001. NS: not significant.

Western blot analysis was employed to investigate the expression of inflammatory cytokines and S1P proteins in the lung tissues of mice groups fed with different diets. The findings reveal a notable downregulation of S1P protein expression in the RSV-infected mice provided with a low-fat diet compared to the control group (Fig. 7A and B). Additionally, the expression levels of inflammatory cytokines such as IL-1β, IL-6, and TNF-α were reduced in the lung tissues (Fig. 7A and B).

### Histological evaluation of inflammation in mouse lung tissue post-different treatments

**Fig 8:**
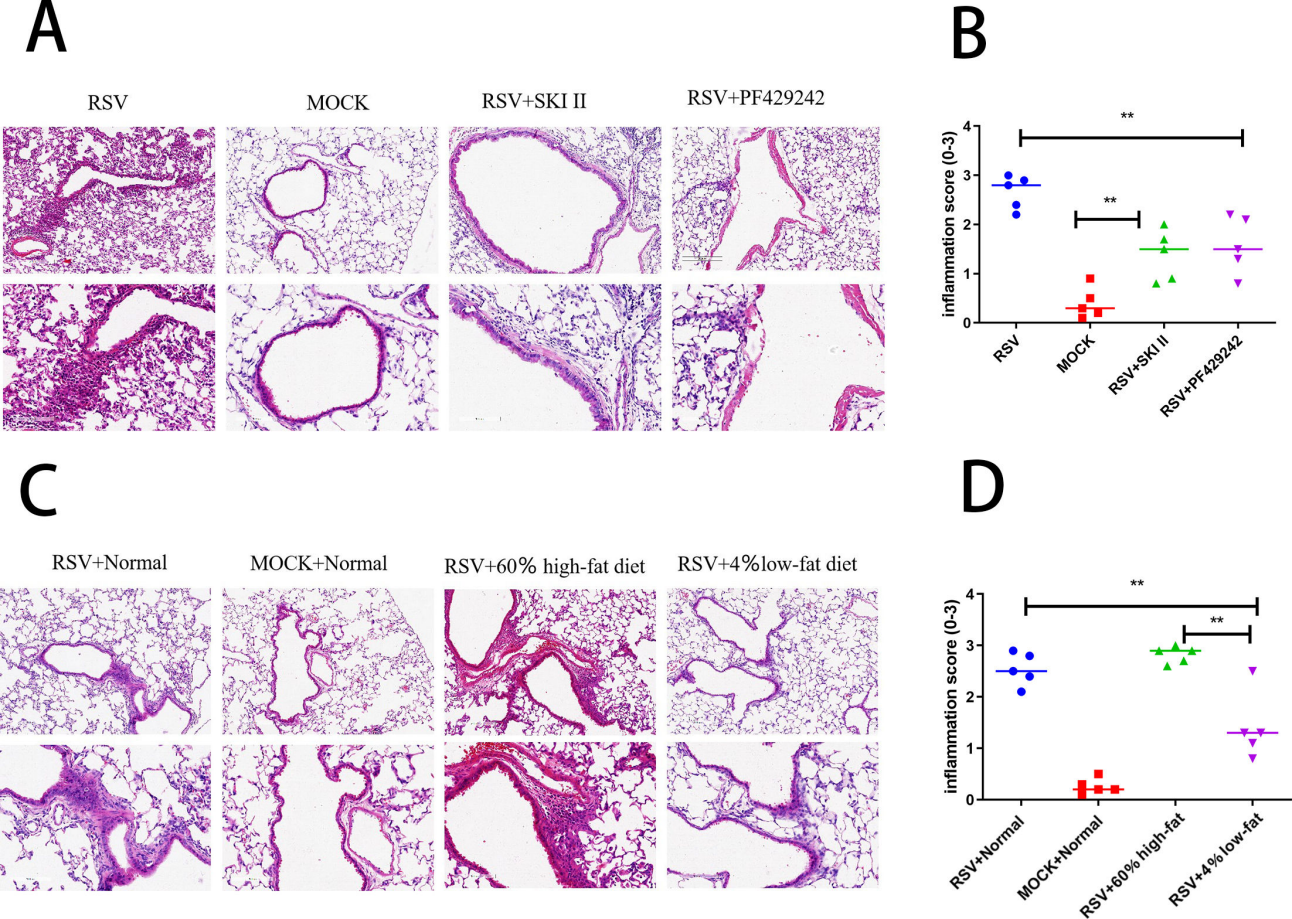
Histological assessment and inflammation scoring of lung tissue in mouse models. (A) HE staining of lung tissues from different groups: RSV-infected mice, control group mice, RSV-infected mice treated with SPHK inhibitor, and RSV-infected mice treated with S1P inhibitor. (B) Inflammation scores for the four groups, as mentioned in A, quantifying the severity of lung tissue inflammation. Markers indicate different groups: blue circles for RSV-infected mice, red squares for control mice, green upward triangles for RSV-infected mice treated with SPHK inhibitor, and purple downward triangles for RSV-infected mice treated with S1P inhibitor. (C) Lung tissue presentation of additional groups: RSV-infected mice, control group mice, RSV-infected mice fed a high-fat diet, and RSV-infected mice fed a low-fat diet. (D) Inflammation scores for the groups in C, comparing the impact of dietary modifications on inflammatory responses in RSV-infected mice. Group markers are the same as described in B: blue circles for RSV-infected mice, red squares for control mice, green upward triangles for RSV-infected mice on a high-fat diet, and purple downward triangles for RSV-infected mice on a low-fat diet. Significance levels: *P < 0.05, **P < 0.01, and ***P < 0.001. NS: not significant.

The application of S1P and SPHK inhibitors in RSV-infected model mice resulted in a reduction of inflammation scoring within lung tissue, as evidenced by HE staining (Fig. 8A and B). Similarly, feeding a low-fat diet to RSV-infected model mice led to a decrease in inflammatory scores in lung tissues (Fig. 8C and D). This suggests that both pharmacological interventions with inhibitors and dietary management can play a role in mitigating pulmonary inflammation in the context of RSV infection.

### Cell counts and differential in bronchoalveolar lavage fluid under different treatments

**Fig 9:**
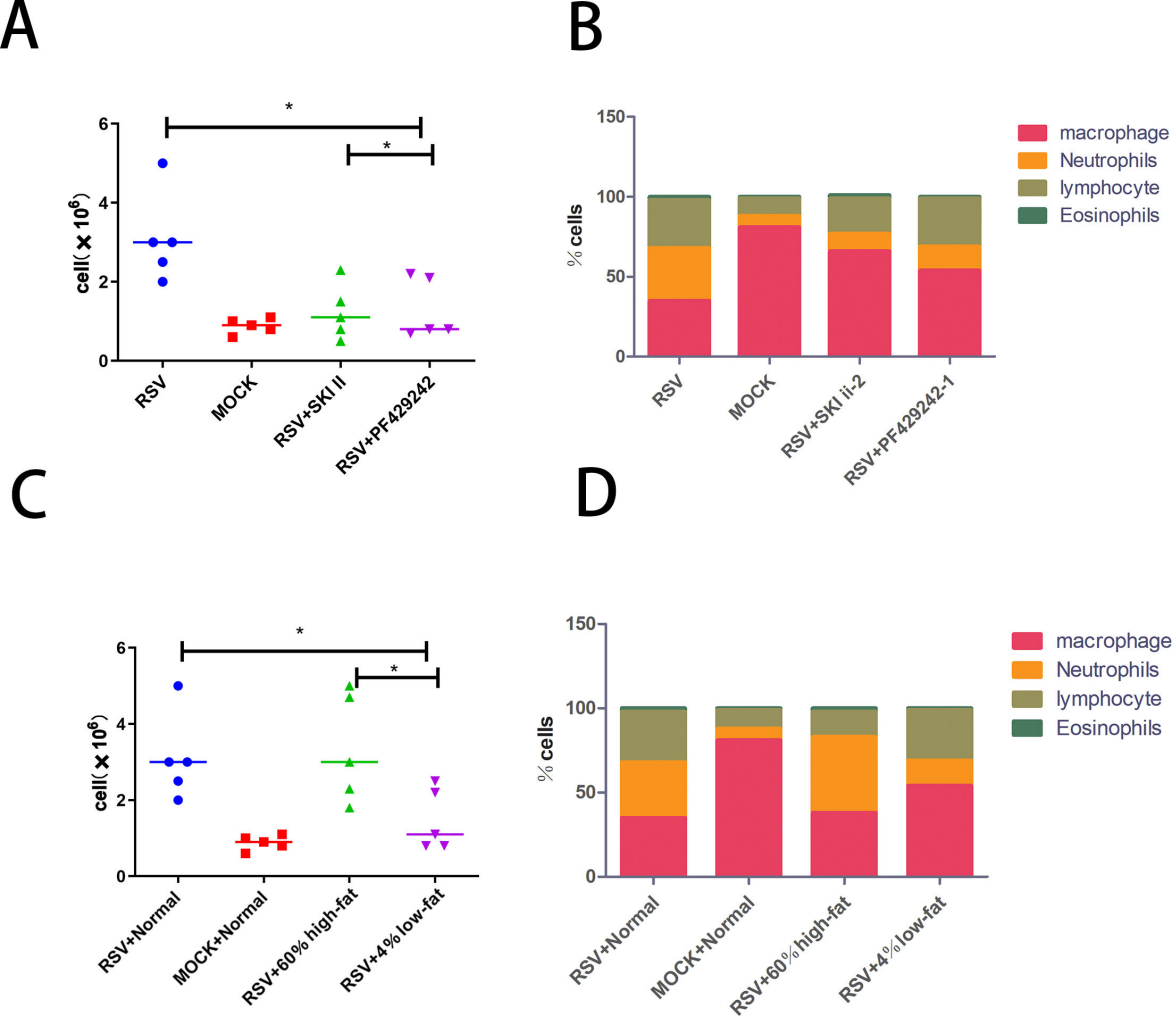
(A) Cell count in BALF after treatment with SPHK and S1P inhibitors in RSV model mice. Markers indicate groups as follows: blue circles for RSV-infected mice, red squares for control mice, green upward triangles for RSV-infected mice treated with SPHK inhibitor, and purple downward triangles for RSV-infected mice treated with S1P inhibitor. (B) Cellular differentiation in BALF after treatment with SPHK and S1P inhibitors in RSV model mice. Group markers are consistent with those in A: blue circles for RSV-infected mice, red squares for control mice, green upward triangles for RSV-infected mice on a high-fat diet, and purple downward triangles for RSV-infected mice on a low-fat diet. (D) Cellular differentiation in BALF after feeding RSV model mice with either a low-fat or high-fat diet. Significance levels: *P < 0.05, **P < 0.01, and ***P < 0.001. NS: not significant.

In the mouse model of RSV infection, the use of SPHK and S1P inhibitors has led to a notable reduction in the total cell count in the bronchoalveolar lavage fluid (BALF). Significantly, the proportion of neutrophils within the BALF was decreased considerably compared to the RSV-infected mice without these treatments (Fig. 9A and B). This suggests that these inhibitors mitigate the inflammatory response in the lungs triggered by RSV. In relation to dietary impacts, when RSV model mice were fed a low-fat diet versus a high-fat diet, it was observed that those on the low-fat regimen showed a reduction in BALF cell counts and a lower proportion of neutrophils compared to the RSV-infected group on a standard diet. Conversely, mice on the high-fat diet exhibited an increase in BALF cell counts and a higher percentage of neutrophils relative to their standard diet counterparts (Fig. 9C and D). These findings indicate that dietary fat content can modulate the inflammatory response in this model of RSV infection.

### Effects of SPHK and S1P inhibitors on lymphocyte subpopulations in lung tissue of RSV-infected mice

**Fig 10:**
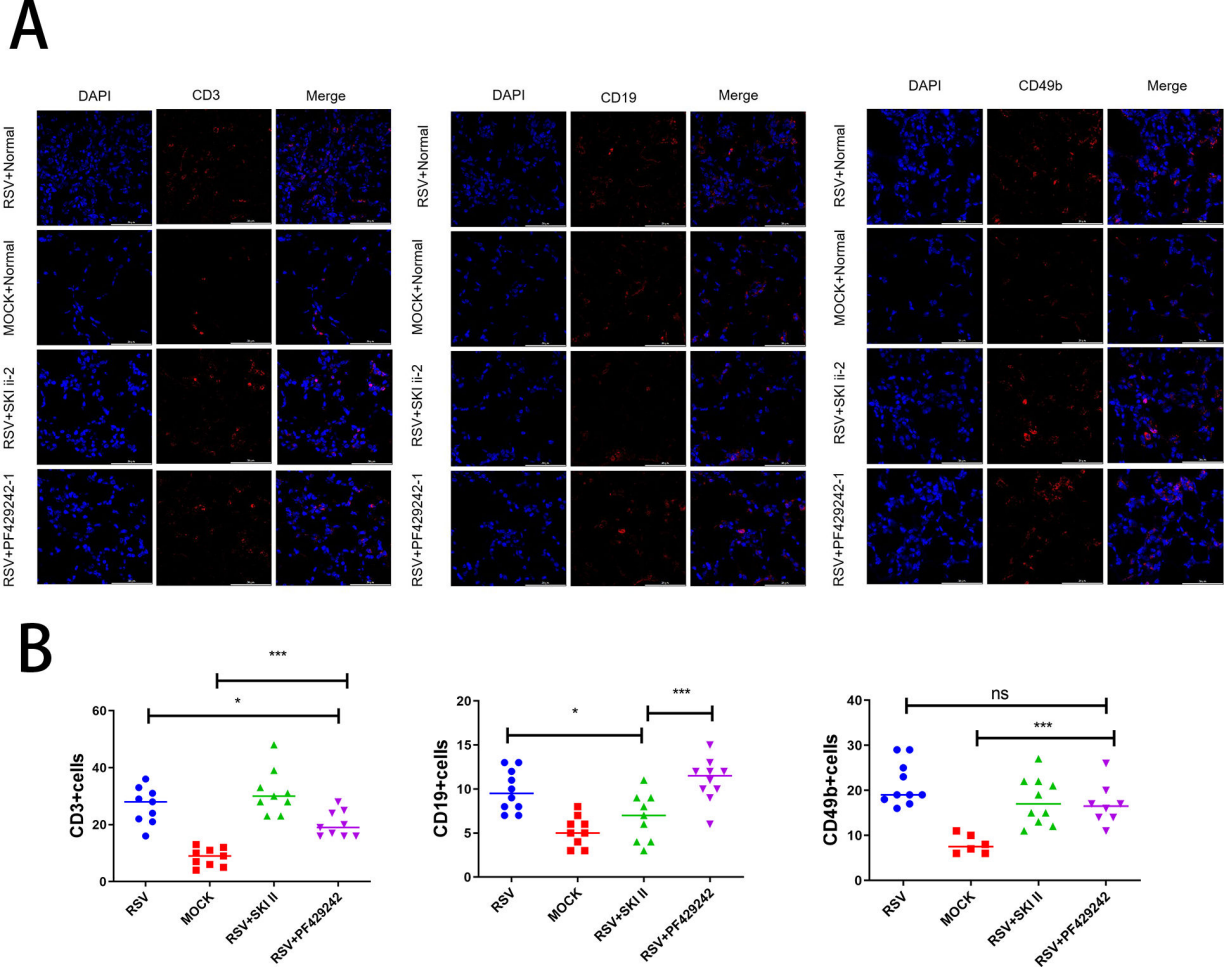
(A) Representative immunofluorescence images of CD19+ B cells, CD3+ T cells, and CD49b+ NK cells in lung tissue sections from control, RSV-infected, and inhibitor-treated groups. (B) Quantitative analysis of lymphocyte subtypes. Markers indicating groups are as follows: blue circles for RSV-infected mice, red squares for control mice, green upward triangles for RSV-infected mice treated with SPHK inhibitor, and purple downward triangles for RSV-infected mice treated with S1P inhibitor. Data are expressed as mean ± SEM (*n* ≥ 6 per group). Significance levels: *P < 0.05, **P < 0.01, and ***P < 0.001. NS: not significant. Note: technical overlap exists between “Mock + Normal” CD3+ images in panel A and Fig. 11A due to consecutive section sampling from the same cohort (see the Methods section on Immunofluorescence Staining of Mouse Lung Tissues).

In the RSV model mice, administration of the SPHK inhibitor resulted in a decreased number of CD19+ B lymphocytes, whereas there were no significant changes in the numbers of CD3+ T lymphocytes and CD49b+ natural killer (NK) cells (Fig. 10A and B). On the other hand, treatment with the S1P inhibitor led to a reduction in the number of CD3+ T lymphocytes, with CD19+ B lymphocytes and CD49b+ NK cells remaining unaffected in their quantities (Fig. 10A and B). These differential effects on lymphocyte subtypes could have implications for understanding how these inhibitors influence immune cell composition in the context of RSV infection. Statistical significance was determined by one-way ANOVA with Tukey’s *post hoc* test (mean ± SEM, *n* ≥ 6).

### Dietary modulation of lymphocyte subpopulations in lung tissue of RSV-infected mice

**Fig 11:**
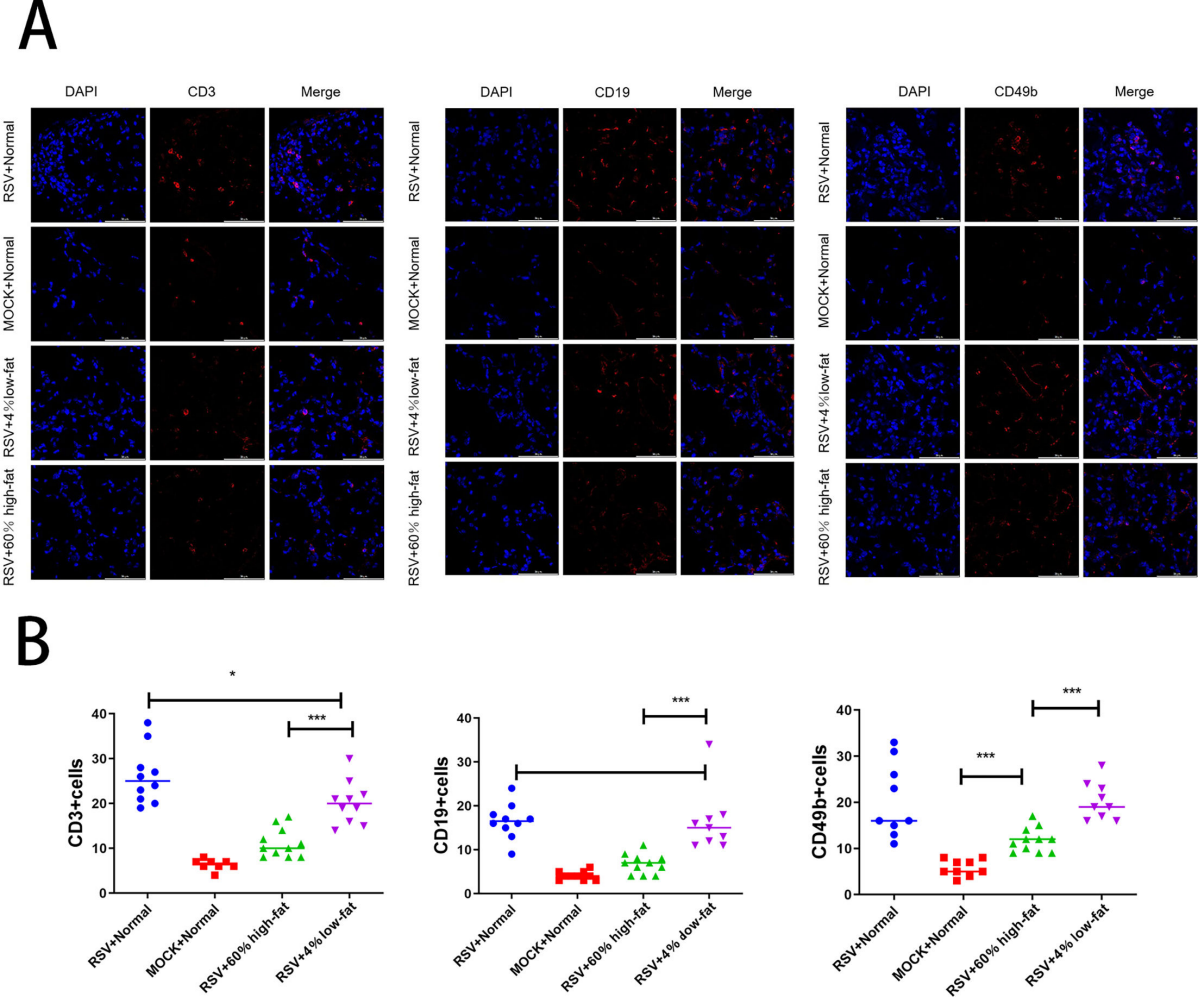
(A) Representative immunofluorescence images showing variations in CD19+ B cells, CD3+ T cells, and CD49b+ NK cells within lung tissue sections of RSV model mice fed high-fat or low-fat diets. (B) Quantitative analysis of lymphocyte subtypes. Group markers are as follows: blue circles for RSV-infected mice, red squares for control mice, green upward triangles for RSV-infected mice on a high-fat diet, and purple downward triangles for RSV-infected mice on a low-fat diet. Data are expressed as mean ± SEM (*n* ≥ 6 per group). Significance levels: *P < 0.05, **P < 0.01, and ***P < 0.001. NS: not significant.

In RSV model mice fed with a low-fat diet, a modest reduction in CD3+ T cells was observed, while no significant changes occurred in the numbers of CD19+ B cells or CD49b+ NK cells (Fig. 11A and B). In contrast, RSV model mice fed a high-fat diet exhibited marked decreases in the numbers of CD3+ T cells, CD19+ B cells, and CD49b+ NK cells (Fig. 11A and B).

## DISCUSSION

To date, there is no conclusive evidence establishing a direct link between the pathogenesis of bronchiolitis and alterations in the gut microbiome. However, some studies suggest that changes in the gut microbiota might impact the immune system and thereby indirectly influence the health of the respiratory system (14, 15). First, the gut microbiota plays a significant role in human health and is closely connected to the development and regulation of the immune system (16). Alterations in the gut microbiota could lead to immune dysregulation, increasing the risk of respiratory infections and inflammation (17), and hence could indirectly affect the incidence of bronchiolitis. Second, the gut-lung axis is hypothesized to be a potential pathological pathway. Microbes and their metabolic byproducts may enter the circulatory system through the gastrointestinal tract and subsequently impact the functions of other organs, including the respiratory system (18). Some studies have found that dysbiosis of the gut microbiome is associated with increased airway inflammation (19). While the relationship between bronchiolitis and changes in the gut microbiota remains controversial, several studies are probing into this area. For example, one study suggests that modulation of the gut microbial composition can alleviate the symptoms of chronic obstructive pulmonary disease (20). Additionally, preliminary studies are also exploring the relationship between the use of antibiotics and respiratory illnesses, as antibiotics can have long-term effects on the gut microbiota and indirectly impact respiratory health. The precise relationship between the gut microbiota and bronchiolitis requires further in-depth research and evidence. More scientific studies are needed in this domain to address the related questions.

Our study sheds new light on how RSV infection impacts host metabolism and reveals the potentially pivotal role of the gut-lung axis in modulating the host’s immune response. Using a mouse model infected with RSV, we observed alterations in the gut microbiome after infection, which correlated with a reduction in the intestinal metabolic product sphingosine and an increase in S1P levels within the lung tissue. Our data suggest that gut-derived metabolic products, particularly sphingosine, may regulate the inflammatory response associated with RSV-induced bronchiolitis through the sphingosine kinase (SPHK)/S1P pathway.

The interaction between the gut and the lungs, often referred to as the gut-lung axis, plays a critical role in regulating the host’s immune and inflammatory responses (21). The composition of the gut microbiota can significantly affect the immune state of the lungs (22), influencing systemic inflammatory responses by altering metabolites that affect distant organs, such as the lungs. Indeed, previous studies have highlighted that dysbiosis of the gut microbiota can impact remote organs, including the lungs, via metabolites disseminated through the bloodstream (23). Our analysis indicates that RSV infection does not significantly alter the host’s gut microbiota’s alpha diversity, aligning with some literature, suggesting that RSV may not directly impact gut microbial diversity. However, this may vary depending on the host’s condition and environmental factors. Despite the overall diversity remaining constant, the composition of the gut microbiota experienced significant changes at both the phylum and genus levels, including increases in Proteobacteria and Bacteroidetes and decreases in Actinobacteria and Firmicutes. These shifts could represent the microecological response to RSV infection, as previously described, and may also indicate underlying microbiome-host interactions between the host gut and distant lung tissue. Further analysis suggests that Proteobacteria spp., specifically the genus Vibrio, may act as key organisms influencing the host’s pathological process. Given the role of Proteobacteria in immune regulation and inflammatory responses (24), their changes could elucidate why certain shifts in phyla distribution are closely tied to specific inflammatory pathways.

Sphingolipids are essential lipids constituting cell membranes and act as secondary messengers involved in numerous cellular signaling pathways, including those regulating cell proliferation, migration, apoptosis, and inflammatory responses (25, 26). In the gut, the synthesis and metabolism of sphingolipids are influenced by the gut microbiota, which in turn may affect the regulation of the host’s immunity (27). In our study, we also observed significant changes at the host metabolite level, particularly concerning the precursors to S1P production, such as sphingomyelin, ceramide, and sphingosine (28), which were found to be significantly reduced in RSV-infected mice compared to healthy controls. ROC analysis results (AUC > 0.7) indicate that these sphingolipid components are reliable biomarkers for differentiating between healthy and RSV-infected mice. These findings are consistent with the role of S1P in regulating immune responses, particularly in inflammation and infection contexts. Notably, an upregulation of S1P protein expression in lung tissue was observed in the RSV-infected mice, suggesting that S1P might play an essential role in the immune response within the lungs. Moreover, the use of SPHK and S1P inhibitors effectively reduced the expression of S1P protein, accompanied by a decrease in the inflammatory cytokines IL-1β, IL-6, and TNF-α, further establishing the central role of the S1P pathway in the inflammatory response.

Our study also observed that a low-fat diet significantly suppresses the expression of the S1P protein in the lung tissue of mice infected with RSV and mitigates inflammation. This finding suggests that dietary habits have a direct impact on the inflammatory response post-infection in hosts, and nutritional adjustments may serve as a potential strategy to alleviate or prevent inflammation following respiratory viral infections. Moreover, our results show a reduction in the cell count of BALF and a decrease in the proportion of neutrophils in mice infected with RSV and fed a low-fat diet, which further supports the notion that diet can modulate immune responses, particularly pulmonary inflammation. In terms of immune cell subpopulations, our research reveals that SPHK inhibitors and S1P inhibitors can specifically affect different immune cell populations, highlighting the significant role of the S1P pathway in the complexity of regulating immune cell functions (29). This insight offers new avenues for the development of targeted therapeutic strategies. Therefore, we propose that a personalized nutritional plan, combined with sphingolipid-targeted therapies, be trialed in high-risk infants during RSV seasons.

Several limitations should be acknowledged. First, our study utilized a murine model, and interspecies differences in gut microbiota composition and sphingolipid metabolism may limit direct extrapolation to humans. Second, the sample size might reduce statistical power to detect subtle microbial shifts. Third, while dietary interventions showed efficacy, the precise mechanisms linking low-fat diets to S1P suppression require further exploration. Future studies should validate these findings in clinical cohorts and investigate whether probiotic or prebiotic interventions can restore sphingolipid homeostasis. In summary, our research provides new insights into how RSV infection affects the host’s gut microbiota, lipid metabolism, and the immune-inflammatory network. The findings demonstrate that dietary modulation and pharmacological intervention of the S1P pathway can mitigate inflammation caused by RSV infection, presenting potential avenues for the development of novel therapeutic strategies to treat RSV infections.

## Data Availability

The data sets presented in this study can be found in online repositories. The repository name and accession number are as follows: NCBI BioProject, accession number PRJNA1017972, publicly released on 2023 Sep 16.
